# Bioaccessibility assessment of stable elements and ^210^Po in food

**DOI:** 10.1371/journal.pone.0236871

**Published:** 2020-08-03

**Authors:** Carla Roselli, Maria Assunta Meli, Ivan Fagiolino, Donatella Desideri

**Affiliations:** 1 Department of Biomolecular Sciences, Università di Urbino Carlo Bo, Urbino (PU), Italy; 2 GRUPPO C.S.A. S.p.A., Rimini (RN), Italy; Chinese Academy of Sciences, CHINA

## Abstract

Element bioaccessibility consists of the fraction of the element that is mobilized from food matrices into digestive extractants. The degree of bioaccessibility of a toxic metal is a fundamental consideration in estimating its bioavailability. In addition, gaining a better understanding of the essential elements released into the gastro intestinal fluids allows a more thorough assessment of the health benefits of food matrices in the field of nutrition science. In the present study, an *in vitro* digestion model simulating gastro-intestinal digestion (GID) was used to investigate the bioaccessibility of stable elements in mixed leaf salad and ^210^Po in various foods (meat, seafood, vegetables). The simulation was carried out over three phases: after a pre-treatment with a saliva solution, raw and cooked seafood samples were subjected to a complete simulated gastrointestinal digestion (gastric digestion followed by bile-pancreas digestion). The ^210^Po bioaccessibility was found to range from 16.2±9.39% to 62.8±17.7% and from 6.26±2.15% to 67.5±13.1% for raw and cooked food respectively. Moreover, bioaccessibility could not be determined for As, Cd, Ce, Co, Cr, Hg, La, Pb, Sb, Sn, Te, Th, Tl, Ti, U. It proved to be poor (1–16%) for Al, Fe and S; fair (40–50%) for Cu, P, and Si; and high (>50%) for Ba, Ca, K, Mg, Mn, Ni, Rb, Sr, Zn. The results show that bioaccessibility varies according to the chemical form of the element in the food as well as the matrix composition.

## Introduction

Any element that is present in the air, or more importantly, in the soil, can be transferred to the crops that are grown in that particular environment. Indeed, for the general population, ingestion of food or beverages is the main pathway through which stable or radioactive contaminants and nutrients are taken into the body.

A high percentage, (about 18% of the average internal dose to the population is due to the ingestion of ^210^Po together with its precursor ^210^Pb) of the natural internal radiation dose attributable to food consumption comes from ^210^Po, a naturally occurring radionuclide of the ^238^U series with a half-life of 138.4 days [1, 2]. Its distribution in the environment depends to a large extent on the other radionuclides in the decay series such as ^226^Ra and ^210^Pb. Most of the natural radioactivity content of plants is accounted for by ^210^Po as the result of the direct deposition of ^222^Rn daughters from atmospheric precipitation, or as a decay product of the ^238^U series in soil. The presence of ^210^Po in all terrestrial foodstuffs is therefore unavoidable [3, 4]. The radiation risk posed by ^210^Po stems from its widespread presence in food, its emission of high energy alpha particles (5,3 MeV), and its affinity for protein. All subjects with high protein diets consisting of meat (primarily reindeer and caribou for Arctic and Subarctic populations) and seafood show elevated levels of ^210^Po [5]. In particular, in population groups whose diet is mainly seafood-based, the consumption of fish can account for a significant fraction of their overall annual dose exposure [6, 7]. The United Nations Scientific Committee on the Effects of Atomic Radiation (UNSCEAR) reports a global average annual ^210^Po intake of 58 Bq [8]. Data collected across five European countries (Italy, Poland, Romania, Russia and the UK) show an average annual ^210^Po dietary intake of 40 Bq [1, 8].

Given that the bioavailabilty of an element is an important datum for the calculation of the internal ingestion dose from intake data and that bioavailability can be equal to or less than bioaccessibility, the more accurate the dissolution data are, the more accurate the calculation of the dose will be.

Regarding stable elements, introducing hazardous substances such as arsenic (As), cadmium (Cd), mercury (Hg), lead (Pb), and antimony (Sb) into the environment [9] poses a serious threat to ecosystems and the health of animals and humans alike [10]. These elements are naturally present in the Earth’s crust and biological systems; however, they accumulate in the environment as a result of industrial and anthropogenic activities. They may then translocate from the environment to humans via the food chain, mainly through the ingestion of plants that are grown in contaminated soils [11–13]. The effects of toxic elements such as Pb, Cd and As on human health is a serious concern since such elements can have adverse effects even at low levels when ingested over a long period [14].

Any assessment of the impact of stable and radioactive toxic metals on human health should consider several different factors [5]. One such factor is the oral bioavailability of the metal, i.e. the fraction of the ingested toxic element in food that enters the bloodstream (absorption factor (f_A_)) [15]. We must also consider the metal distribution from the blood to the various organs as well as its retention time in the body. To make accurate dose assessments of ingested polonium we must have realistic estimates of f_A_ values for different food items. In addition, gaining a better understanding of the essential elements released into gastro intestinal fluids is important in the field of nutrition in order to evaluate the health benefits of food matrices. Moreover, the assessment of nutrient bioavailability in specific sites of the human gastrointestinal tract is also important in food technology for the development of a new generation of structured foods specifically designed to maximize their health benefits.

*In vitro* digestion models that assess bioaccessibility represent an alternative to *in vivo* experiments. *In vitro* models simulate the concentration, temperature and pH of digestive juices as well as the transit time of food in the digestive process, making it possible to assess the degree of mobilization of the contaminant or nutrient from a food matrix into digestive extractants. Bioaccessibility is the necessary condition for a contaminant or nutrient to become bioavailable. Moreover, bioaccessibility, which may be sensitive to matrix effects, can be considered as an indicator of maximal oral bioavailability [16]. The total bioavailability (f_A_) of any element or compound is therefore the result of the following three factors: a) bioaccessibility, b) the degree of transport across the intestinal epithelium and c) the unmetabolized fraction that passes through the liver [17]. If we lack accurate information on bioaccessibility and, consequently, on oral bioavailability, we may over or underestimate the risks posed by a food contaminant or the potential benefits deriving from the release of nutrients from a given food. The literature provides little information regarding the bioaccessibility of radioactive and stable elements in various food items tested using *in vitro* digestion models [18–25].

In the present study the authors have continued to pursue lines of research initiated in previous works [26, 27]. In those works, the mobilization of ^210^Po from seafood and toxic metals from algae was investigated by analyzing separate fractions obtained after contact with solutions that simulated the chemical environment of the stomach (acidic) and bile-pancreas (alkaline), employing an *in vitro* digestion model proposed by Oomen et al. [17]. The present investigation focuses on the mobilization of polonium and stable elements from different food matrices such as meat, seafood, and vegetables.

## Materials and methods

### Samples and sample pretreatment

Seven samples representing three different categories of food were selected for the study: three meat samples (beef, turkey, horse), two seafood samples (fish: anchovy, *Engraulis encrasicolus*, crustacean: mantis shrimp, *Squilla mantis*), two vegetable samples (several varieties of homegrown or store-bought mixed leaf salad) (Table 1). The analyzed samples were purchased from large-scale retailers and shops is the township of Urbino (Central Italy). Twenty individual specimens were selected for each category of seafood sample to obtain a single bulk sample. Meat and seafood samples were first washed, then muscle (fish), or edible parts (crustaceans) were removed, homogenized and weighed. Subsequently, 100 g of each bulk sample was boiled for 5 minutes at 100°C in 500 ml of water. The sample was then filtered to remove the cooking water. Finally, the cooked samples as well as the untreated samples (raw samples) were frozen at -20° C. The following day, the samples were freeze-dried for 24 h using an Edwards modular freeze dryer.

**Table 1:** Ratio between raw and cooked weight and ratio between raw or cooked weight and lyophilized weight in seven food samples.

	Sample	Raw weight cooked weight	Cooked weight dry weight	Raw weight dry weight
**Meat**	beef	1.46	2.91	4.25
	turkey	1.43	2.67	3.82
	horse	1.51	2.57	3.88
	***mean±SD***	***1*.*47±0*.*04***	***2*.*72±0*.*17***	***4*.*00±0*.*23***
**Seafood**	anchovy	1.50	3.62	5.43
	mantis shrimp	1.12	4.90	5.49
	***mean±SD***	***1*.*31±0*.*27***	***4*.*26±0*.*91***	***5*.*46±0*.*04***
**Vegetable**	mixed leaf salad from vegetable garden	-	-	15.6
	mixed leaf salad from supermarket	-	-	23.8
	***mean±SD***			***19*.*7±5*.*8***

All mixed leaf salad samples (both those purchased from large-scale retailers and from growers) were washed thoroughly with running water and the inedible parts were discarded. After being dried, the samples were frozen at -20°C and lyophilized under the same conditions as the meat and seafood products.

The dehydrated samples were then weighed and homogenized. Table 1 shows the ratio between raw and cooked weight, ranging from 1.43 to 1.51 and from 1.12 to 1.50 for meat and seafood respectively. These variations are due to the variable water loss during cooking. Table 1 also shows the ratio between cooked weight and lyophilized weight, which ranged from 2.57 to 2.91 and from 3.62 to 4.90 for meat and seafood respectively. The ratio between raw weight and lyophilized weight ranged from 3.82 to 4.25, 5.49 to 5.43 and 15.6 to 23.8 for meat, seafood and vegetables, respectively.

### Complete dissolution

Samples were completely dissolved to compare the total quantity of ^210^Po and stable metals contained in each sample with the amount of those metals leached into human fluids.

#### Chemical procedure of ^210^Po

^210^Po preparation and dissolution were carried out by the addition of a known activity of ^209^Po tracer to 1–2 g of every sample (raw or cooked).

The sample was then subjected to wet digestion in hot concentrated nitric acid (69% w/v) and hydrogen peroxide (30% v/v) in a closed glass reactor heated to 200°C. The loss of polonium should be limited by these conditions. The solution was subsequently evaporated to dryness, and the residue was retreated several times with concentrated nitric acid and hydrogen peroxide. Once a clear solution had formed (after about 15 h), all the remaining nitric acid was evaporated, and concentrated HCl was added in order to convert the nitric acid to a chloride form. Lastly, the residue was redissolved in 50 mL of 1M HCl and the solution was then filtered [13].

#### Stable elements

The sample (1–1.5 g) was dissolved using the 3050B method (1996) proposed by the Environmental Protection Agency [28] and described in detail by Meli et al. [29]. The method involves the following principal steps: a) addition to the sample a mixture of 69% w/v nitric acid, 37% w/v hydrochloric acid and 30% v/v hydrogen peroxide; b) reflux treatment for 1 h at 95°C in the block digester; c) filtration and washing d) sample collection obtaining a 1% solution.

#### Digestion procedures

The *in vitro* digestion model is a useful alternative to animal and human models allowing the rapid screening of food ingredients [30]. The technique is reproducible, reliable and sensitive to changes due to processes. Moreover, it is faster, less complicated and less expensive than *in vivo* measurements [19, 21, 22]. A number of *in vitro* digestion models are currently available for assessments in different fields of study (nutrition, toxicology etc.). In the present study, the simulation of gastrointestinal digestion was based on the dynamic model reported by Oomen et al. [17]. A part of each sample, already used for complete dissolution, was subjected to a complete gastrointestinal digestion (GID). Digestive juices (salivary, gastric, duodenal and biliary juices), whose compositions were based on human physiology, were prepared artificially (Table 2). The detailed procedures have been described in the authors’ previous studies [26, 27, 31]. The plastic bottle containing the sample and the artificial digestive fluids was kept in a thermostatic room at 37± 2°C, the normal temperature of the human body (enzyme activity and chemical characteristics such as solubility are affected by temperature) to simulate the conditions of the human digestive tract. The sample and digestive fluids were mixed constantly by placing the bottle in a rotator at a speed of 55 rpm; the bottles were rotated head-over-heels. The volumes (ml) and quantities (mg) of the extractants used for 1 g of the sample as well as the schematic procedures of these simulations are shown in Table 3.

**Table 2:** Constituents, relevant concentration of 1 L of salivary, gastric, duodenal and biliary juices [17].

Constituent	Concentration (g/L)	Salivary juice (ml)	Gastric (ml)	Duodenal (ml)	Biliary (ml)
**NaCl**	175.3	1.7	15.7	40	30
**KCl**	89.6	10	9.2	6.3	4.2
**KSCN**	20.0	10			
**NaH**_**2**_**PO**_**4**_	88.8	10	3.0		
**Na**_**2**_**SO**_**4**_	57.0	10			
**KH**_**2**_**PO**_**4**_	8.0			10	
**NaHCO**_**3**_	84.7			40	68.3
**NaOH**	40.0	1.8			
**CaCl**_**2**_	22.2		18	9.0	10
**MgCl**_**2**_	5.0			10	
**NH**_**4**_**Cl**	30.6		10		
**HCl**	440.3		8.3	0.5	0.5
**Urea**	25.0	8	3.4	4.0	10
**glucose**	65.0		10		
**glucoronic acid**	2.0		10		
**glucosamine hydrochloride**	33.0		10		
**mucin**		50 mg	3000 mg		
**α-amylase**		145 mg			
**uric acid**		15 mg			
**BSA**			1000 mg	1000 mg	1800 mg
**pepsin**			1000 mg		
**pancreatin**				3000 mg	
**lipase**				500 mg	
**bile**					6000 mg

**Table 3:** Complete digestion procedures for 1 g of the sample at 37°C (the volumes of the extractants were multiplied by two, three or four depending on the amount of sample, 1–4 g) (Desideri et al. 2018a).

Digestion	Samples	Extraction reagents	Time, min
1° stage	1 g	0.6 ml salivary solution pH = 6.5±0.2	5
2° stage	resulting suspension after the 1° stage	1.33 ml gastric solution pH = 1.07±0.07	120
3° stage	resulting suspension after the 2° stage	1.33 ml duodenal solution and 0.67 ml biliary solution pH = 8.0±0.2	120

After each treatment, the suspension that was obtained was centrifuged at 12,000 rpm for 30’ to separate the digestive juice from the digested sample. The solution was then evaporated to dryness, and the residue was dissolved in 1M HCl before being filtered. The solution was subsequently divided into two parts: one for polonium analysis (a known activity of ^209^Po was added as an internal yield standard) and one for the analysis of stable elements. The gastrointestinal digestion was performed three times to test the reproducibility of the method. Finally, the mean of the results obtained in the two sequences was calculated. In addition, a blank sample was prepared to account for the possible impurity of reagents and spillage from containers.

### ^210^Po and elemental analysis

The ^210^Po determination and elemental analysis were carried out in the solution originating from the complete dissolution and digestion procedure.

#### ^210^Po determination

Alpha spectrometry was used to assess ^210^Po. The determination method includes a source preparation and measurement.

Ten ml of 20% hydroxylamine hydrochloride and 10 ml of 25% sodium citrate were added to the solutions derived from the complete dissolution or from the digestion procedure. Both ^210^Po and ^209^Po were plated continuously at 85–90°C and 1.5–2.0 pH on a silver disk for 4 h. They were then placed in a syringe and immersed in the solution derived from the complete dissolution of the sample or from the digestion procedures (200 mL of 1 M HCl). No preliminary separation was necessary, and essential quantitative recoveries were calculated using the ^209^Po tracer [32]. The discs were counted by an α-spectrometer fitted with a silicon surface barrier semiconductor detector (300 mm^2^ active surface, resolution 20 keV, 31.7±3.1% counting efficiency, and 2 x 10^−6^ s^-1^ of the background in the affected energy region) (Canberra Industries, Inc., 800 Research Parkway, Meriden, CT 06450) and connected to a computerized multichannel analyzer. To achieve reliable counting statistics counting times ranged from 1000 to 3000 min. The activity concentration of ^210^Po was calculated by drawing a comparison between the areas of the two clearly resolved peaks of ^210^Po (at 5.30 MeV) and ^209^Po (at 4.88 MeV). The mean chemical yield for polonium was 88.1±9%, and the Minimum Detectable activity Concentration (MDC) was 0.007 Bq·kg^-1^.

#### Elemental analysis

The solutions obtained from sample dissolution were analyzed using three different methods. Inductively Coupled Plasma-Atomic Emission Spectrometry (ICP-AES) was used for Al, As, Ba, Ca, Cd, Co, Cr, Cu, K, Mg, Mn, Ni, P, S, Si, Sr, Ta, Th, Ti and Zn, while Inductively Coupled Plasma-Mass Spectrometry (ICP-MS) was used for Ce, La, Rb, and U. The methods were applied as described in EPA 6010D 2014 [33] and EPA 6020B 2014 [34] respectively. ICP-MS and ICP-AES are the instrumental methods of choice for determining trace elements in plant tissues [11]. Indeed, ICP-MS and ICP-AES are multielement techniques that offer a wide range of analytical applications, extended dynamic concentration ranges, high selectivity and sensitivity and low analytical limits. Hg was determined by EPA 7470A [35] using SpectrAA220 Fast Sequential, Varian, Palo Alto, CA (thermal decomposition, amalgamation and atomic absorption spectrometry [36]). The MDC of every element that was determined are reported in Table 4.

**Table 4:** Element determination methods for solid and liquid matrices with relative minimum detectable concentrations (MDC).

Element	Method for solid matrix	MDC (mg/kg_ww_)	Method for liquid matrix	MDC (mg/L)
**Aluminum**	EPA 3050 1996 + EPA 6010D 2014	0.163	EPA 6020B 2014	0.005
**Antimony**	EPA 3050 1996 + EPA 6010D 2014	0.006	EPA 6020B 2014	0.001
**Arsenic**	EPA 3050 1996 + EPA 6010D 2014	0.006	EPA 6020B 2014	0.001
**Barium**	EPA 3050 1996 + EPA 6010D 2014	0.160	EPA 6020B 2014	0.0005
**Cadmium**	EPA 3050 1996 + EPA 6010D 2014	0.0003	EPA 6020B 2014	0.001
**Calcium**	EPA 3050 1996 + EPA 6010D 2014	32.05	EPA 6020B 2014	0.5
**Cerium**	EPA 3050 1996 + EPA 6020B 2014	0.064	EPA 6020B 2014	0.001
**Chromium**	EPA 3050 1996 + EPA 6010D 2014	0.032	EPA 6020B 2014	0.001
**Cobalt**	EPA 3050 1996 + EPA 6010D 2014	0.032	EPA 6020B 2014	0.001
**Copper**	EPA 3050 1996 + EPA 6010D 2014	0.032	EPA 6020B 2014	0.001
**Iron**	EPA 3050 1996 + EPA 6010D 2014	0.160	EPA 6020B 2014	0.005
**Lanthanium**	EPA 3050 1996 + EPA 6020B 2014	0.064	EPA 6020B 2014	0.001
**Lead**	EPA 3050 1996 + EPA 6010D 2014	0.006	EPA 6020B 2014	0.001
**Magnesium**	EPA 3050 1996 + EPA 6010D 2014	0.032	EPA 6020B 2014	0.5
**Manganese**	EPA 3050 1996 + EPA 6010D 2014	0.032	EPA 6020B 2014	0.001
**Nickel**	EPA 3050 1996 + EPA 6010D 2014	0.032	EPA 6020B 2014	0.0005
**Phosphorus**	EPA 3050 1996 + EPA 6010D 2014	0.032	EPA 200.7 2001	0.01
**Potassium**	EPA 3050 1996 + EPA 6010D 2014	32.05	EPA 6020B 2014	0.5
**Rubidium**	EPA 3050 1996 + EPA 6020B 2014	0.064	EPA 6020B 2014	0.001
**Silicon**	EPA 3050 1996 + EPA 6010D 2014	0.641	EPA 200.7 2001	0.01
**Strontium**	EPA 3050 1996 + EPA 6010D 2014	0.032	EPA 6020B 2014	0.001
**Sulfur**	EPA 3050 1996 + EPA 6010D 2014	0.064	EPA 200.7 2001	0.1
**Tellurium**	EPA 3050 1996 + EPA 6010D 2014	0.032	EPA 6020B 2014	0.001
**Thallium**	EPA 3050 1996 + EPA 6010D 2014	0.006	EPA 6020B 2014	0.001
**Thorium**	EPA 3050 1996 + EPA 6020B 2014	0.006	EPA 6020B 2014	0.0025
**Tin**	EPA 3050 1996 + EPA 6010D 2014	0.032	EPA 6020B 2014	0.001
**Titanium**	EPA 3050 1996 + EPA 6010D 2014	0.032	EPA 200.7 2001	0.001
**Uranium**	EPA 3050 1996 + EPA 6020B 2014	0.064	EPA 6020B 2014	0.001
**Zinc**	EPA 3050 1996 + EPA 6010D 2014	0.032	EPA 6020B 2014	0.005
**Mercury**	EPA 7473 2007	0.00003	EPA 6020B 2014	0.0001

### Quality control

For the analysis of stable and radioactive elements, it is necessary to prepare a blank sample to check for any possible contamination with impurities from reagents, containers or instrumentation. The blank sample was obtained by mixing all the reagents and applying the same procedures without adding the sample. The accuracy of the method for the determination of stable elements was assessed using a Laboratory Control System (LCS) in which spike recovery tests were performed on a blank sample to which known quantities of analytes (reference sample) were added. The mean results obtained by replicate preparation and analysis of reference samples (NIST 1575A trace elements in pine needles) showed a 20% difference from the expected values (analytical standard errors). The uncertainties of all ^210^Po measurements were calculated taking into consideration the statistical fluctuations of peaks and backgrounds, and efficiency calibration. The accuracy of the radioanalytical method was verified regularly by the authors’ participation in periodic intercomparison exercises organized by the International Atomic Energy Agency (IAEA). The mean analytical standard errors were below 10% compared to the certified activity concentrations for reference materials. Regarding the reproducibility and precision of the methods of chemical and radiological characterization, replicated analyses were performed on the same sample, and the average deviation was less than 10%.

## Results

### Concentrations of ^210^Po and stable elements

The mean values of the ^210^Po activity concentrations in raw food, shown in Table 5, were 0.044±0.019, 28.1±3.86 and 0.054±0.026 Bq/kg for meat, seafood and vegetables, respectively; the highest activity concentration (29.9±3.90 Bq/kg) was found in the mantis shrimp sample. The mean values of the ^210^Po activity concentrations in cooked food were 0.104±0.039 and 26.3±2.34 Bq/kg for meat and seafood respectively; the highest activity concentration (32.7±2.43 Bq/kg) was found in the anchovy sample.

**Table 5:** ^210^Po activity concentrations (mean of three analyses) in seven food samples with relative standard deviations (SD); 210Po activity concentrations (Bq/kg) with relative standard deviations (SD) on three data points in gastro-intestinal juices (GID) for raw (vegetable) and cooked (meat and seafood) samples and ^210^Po extraction %) (bioaccessibility).

	Sample	Raw food Bq/kg	Cooked food Bq/kg	Raw food GID Bq/kg	Cooked food GID Bq/kg	Raw food GID %	Cooked food GID%
**Meat**	Beef	0.056 ± 0.004	0.129 ± 0.057		0.0086±0.0027	15.43±4.866	6.630±2.090
	Turkey	0.021 ± 0.002	0.074 ± 0.038		0.0049±0.0024	23.71±11.73	6.583±3.256
	Horse	0.056 ± 0.016	0.092 ± 0.027		0.0052±0.0010	9.386±1.725	5.650±1.039
	***mean±SD***	***0*.*044 ± 0*.*019***	***0*.*104 ± 0*.*039***		***0*.*0060±0*.*0025***	***16*.*24±9*.*391***	***6*.*256±2*.*147***
**Seafood**	Anchovy	26.3 ± 3.56	32.7± 2.43		20.4± 1.37	77.46±5.194	62.35±3.256
	Mantis shrimp	29.9 ± 3.90	19.8 ± 1.93		14.4 ± 3.49	48.16±11.67	72.68±17.61
	***mean±SD***	***28*.*1 ± 3*.*86***	***26*.*3 ± 2*.*34***		***17*.*4 ± 4*.*04***	***62*.*81±17*.*75***	***67*.*51±13*.*07***
**Vegetable**	Homegrown mixed leaf salad	0.076 ± 0.015	-	0.028±0.013		35.95 *±*17.56	
	Store-bought mixed leaf salad	0.031 ± 0.002	-	0.016±0.040		51.91±13.89	
	***mean±SD***	***0*.*054 ± 0*.*026***	**-**	***0*.*022±0*.*009***		***46*.*59±15*.*67***	

The non-essential or toxic elements including As, Ce, La, Sn, Te, Th, Tl, Ti, U were consistently below their MDC (Table 6); Cd, Hg and Pb ranged from 0.002 to 0.0256 mg/kg_ww_; Ba, Ni, Rb and Sb ranged from 0.147 to 0.654 mg/kg_ww_; Al and Sr concentrations were 1.93 and 1.83 mg/kg_ww_ respectively. Regarding essential elements, the Co concentration was below the MDC. Cr and Cu concentrations were 0.173 and 0.474 mg/kg_ww_ respectively; Fe, Mn, Si and Zn ranged from 1.92 to 8.20 mg/kg_ww_; Ca, Mg, P and S ranged from 89.9 to 434 mg/kg_ww;_ potassium showed the highest concentration at 2333 mg/kg_ww_.

**Table 6:** Element concentrations (mg/kg_ww_) (mean of three replicates) in one sample of homegrown mixed leaf salad, element % extraction in two gastrointestinal digestion replicates (GID1 and DIG2), mean and relative standard deviation (SD).

	Element	mg/kg_ww_	GID_1_%	GID_2_%	Mean	SD
**Non essential**	Aluminum	*1*.*93*	*6*.*84*	*5*.*25*	*6*.*04*	*1*.*13*
**or toxic**	Antimony	*0*.*147*	*<0*.*68*	*<0*.*68*	-	-
	Arsenic	<0.006	ND	ND	ND	ND
	Barium	*0*.*654*	*>100*	*>100*	*>100*	*-*
	Cadmium	*0*.*0020*	*<50*.*0*	*<50*.*0*	-	-
	Cerium	<0.064	ND	ND	ND	ND
	Lanthanium	<0.064	ND	ND	ND	ND
	Lead	*0*.*0256*	*<3*.*90*	*<3*.*90*	-	-
	Mercury	*0*.*0038*	*<26*.*0*	*<26*.*0*	-	-
	Nickel	*0*.*147*	*>100*	*89*.*3*	*>100*	*-*
	Rubidium	*0*.*641*	*95*.*2*	*85*.*9*	*90*.*6*	*6*.*54*
	Strontium	*1*.*83*	*81*.*2*	*88*.*4*	*84*.*8*	*5*.*04*
	Tellurium	<0.032	ND	ND	ND	ND
	Thallium	<0.006	ND	ND	ND	ND
	Thorium	<0.006	ND	ND	ND	ND
	Tin	<0.032	ND	ND	ND	ND
	Titanium	<0.032	ND	ND	ND	ND
	Uranium	<0.064	ND	ND	ND	ND
**Essential**	Calcium	*434*	*>100*	*>100*	*>100*	*-*
	Cobalt	<0.032	ND	ND	ND	ND
	Chromium total^(1)^	0.173	*<0*.*68*	*<0*.*68*	-	-
	Copper	*0*.*474*	*30*.*4*	*66*.*6*	*48*.*5*	*25*.*6*
	Iron	*4*.*02*	*1*.*03*	*2*.*22*	*1*.*62*	*0*.*84*
	Magnesium	*89*.*9*	*99*.*6*	*>100*	*>100*	*-*
	Manganese	*1*.*92*	*90*.*8*	*99*.*8*	*95*.*3*	*2*.*11*
	Phosphorus	*313*	*31*.*1*	*55*.*1*	*43*.*1*	*17*.*0*
	Potassium	*2333*	*>100*	*>100*	*>100*	*-*
	Silicon	*8*.*20*	*40*.*2*	*39*.*5*	*39*.*8*	*0*.*521*
	Sulfur	*127*	*9*.*08*	*23*.*2*	*16*.*2*	*10*.*0*
	Zinc	*2*.*55*	*>100*	*>100*	*>100*	*-*

ND = Not detectable.

(1) = Cr ^6+^ + Cr^3+^

### ^210^Po and stable element bioaccessibility

The mean concentrations of ^210^Po activity (Table 5) were obtained by repeating the digestion procedure three times for both raw (vegetable) and cooked foods (meat and seafood). In addition, the table shows the extraction percentage of polonium in gastro-intestinal digestion (^210^Po bioaccessibility).This was calculated from the ratio between the activity of ^210^Po in the digestive fluids and in the food before digestion: Bq (in digestive juice) x 100/ Bq (in cooked or raw food). For meat and seafood samples, the bioaccessibility in raw samples was also calculated taking into account the raw and cooked weight ratio (Table 1).

The bioaccessibility of stable elements from a raw sample of mixed leaf salad (Table 6) was not calculated for As, Ce, Co, La, Sn, Te, Th, Tl, Ti, or U because their concentrations were below the MDC (Table 4) in the sample; the calculation of the extraction % was performed using the MDC value for Sb, Cd, Po and Hg in the toxic elements and Cr in the essential elements. It was possible to determine the concentrations of all the elements in the sample as such but not in the digestive juices.

## Discussion

The data show that the bioaccessibility (GID%) of ^210^Po varied among the analyzed samples; however, it was consistently lower in meat and higher in seafood products, regardless of whether they were raw or cooked. For meat, the bioaccessibility of polonium would seem to be even lower in the cooked sample. This is probably due to the loss of the most soluble form of the radionuclide in the water during cooking.

The value of the f_A_ factor, determined by calculating the difference between polonium intake and its faecal elimination [4, 6, 37] in animal and human models, ranged from 0.1 [38] to 0.5 [39]. The latter value is still used to calculate the internal digestion dose. From the data obtained in the present study using an *in vitro* digestion model, which are consistent with data from a previous investigation carried out by the authors [31], the following conclusions can be drawn: a) the ^210^Po bioaccessibility values that were obtained do not differ greatly from those obtained using *in vivo* tests; b) the amount of polonium dissolved in the digestive juices seems to vary with variations in food type. This is probably due to the chemical form of the element in the food and the chemical-physical characteristics of the food matrix.

The bioaccessibility of stable elements was only assessed in one uncooked sample of mixed leaf salad that had been purchased directly from a grower. The following essential elements were found to be particularly soluble in the digestive juices (extraction > 100%): Ca, Mg, Mn, K, Zn; Cu, P and Si were moderately bioaccessible (40–50% extraction). This appears to confirm the important role of mixed leaf salad as a source of elements that are essential to the proper functioning of the human body. Cr, Fe, S were not very mobile (extraction < 0,7–16%). Co was less than the MDC even in the sample as such.

The following toxic elements were below the MDC in the mixed leaf salad sample as such and their degree of dissolution could not be calculated: As, Ce La, Te, Tl, Th, Sn, Ti and U. On the other hand, Ba Ni, Ru and Sr were almost completely bioaccessible (85–100% extraction), while Al, Cd, Pb, Hg were found to be poorly soluble (extraction <0,7-<50%). This finding would appear to be reassuring as regards the consumption of mixed leaf salad because although the sample was found to have detectable concentrations of toxic elements, in vitro digestion revealed their poor bioaccessibilty and consequent poor bioavailability. There is limited data in the literature on the bioaccessibility of stable elements from vegetable sources, while the dissolution of stable elements in seafood has been studied much more thoroughly [24, 25, 40–42]. In any case, the results of the present investigation suggest that: a) the digestive juices produce critical changes in the bulk composition of the sample leading to the dissolution of some toxic and essential elements, which migrate from the solid to the digestive tract; b) the content of stable elements in digestive juices would seem to vary with the chemical form and the speciation of the element, but this must be confirmed in further investigations that the authors intend to pursue focusing on other vegetable and animal-based food products.

## Conclusions

In the present study, the degree of mobilization of ^210^Po, toxic and essential elements from three different categories of food matrices into the digestive extractants (bioaccessibility) was assessed. The following three-stage *in vitro* digestion model reported in the literature was used: after a pre-treatment with a saliva solution, raw and cooked seafood samples were subjected to a simulated complete gastrointestinal digestion (gastric digestion followed by bile-pancreas digestion).

The results show that the percentage of the elements dissolved in the digestive juices seems to vary with variations in the food type. This is probably due to the chemical form of the element in the food and the chemical-physical characteristics of the food matrix. Moreover, the accuracy of the value of the absorption fraction is of crucial importance since it affects the dose estimate of the ingested toxic element. As far as the essential elements are concerned, gaining insights into the nutrients released into gastro intestinal fluids is important in the field of nutrition to evaluate the health benefits of a food matrix. Moreover, the assessment of the nutrient bioavailability in specific sites of the human gastrointestinal tract is also important in food technology for the development of a new generation of structured foods specifically designed to improve human health.
